# Early effects of the COVID-19 pandemic on the acute flaccid paralysis surveillance in East and Southern African countries

**DOI:** 10.11604/pamj.2021.39.147.28884

**Published:** 2021-06-24

**Authors:** Daudi Manyanga, Brine Masvikeni, Marybennah Kuloba, Charles Byabamazima, Fussum Daniel

**Affiliations:** 1 WHO Inter-Country Support Team office for East and Southern Africa, Harare, Zimbabwe

**Keywords:** COVID-19 pandemic, lockdown, social distancing, surveillance, poliovirus

## Abstract

**Introduction:**

the World health organisation (WHO) African Region reported the first confirmed COVID-19 case caused by the SARS-CoV-2 on 25^th^ February 2020, and the first case for the East Southern Africa (ESA) sub-region was on 5^th^ March 2020. Almost all countries in the ESA sub region implemented the WHO-recommended preventive measures variably after the notification of community transmission of the COVID-19 disease. This resulted in the disruption of the outpatient, immunization surveillance, and the related supply chain activities.

**Methods:**

a comparative analysis study design of secondary acute flaccid paralysis (AFP) surveillance data received from the East and Southern Africa sub-region countries to evaluate the effect of the COVID-19 pandemic in the AFP field surveillance for the same time period of March to December 2019 and 2020.

**Results:**

we observed that 52.4% of second stool samples were received in the laboratory within 72 hours from March to December 2019, and only 48.1% in the same period of 2020. A 4.3% decline with a p-value of <0.0001 (95% CI, ranges from 2.326% to 6.269%). Similarly, we noted a 4.7% decline in the number of reported AFP cases in the ESA sub-region for March to December 2020 compared to the same period in 2019, a p-value of less than 0.001 (95% CI ranges from 2.785 to 6.614). For the percentage of stool adequacy, we observed a 3.37% decline for April in 2020 compared to April 2019 with a p-value of less than 0.001 (95% CI ranges from 2.059 to 4.690).

**Conclusion:**

we observed a decline in the core AFP surveillance (non polio) NP-AFP rate, and percentage of stool adequacy in countries severely affected by the COVID-19 disease. These countries implemented stringent transmission prevention measures such as lock-down and international transportation restrictions.

## Introduction

Poliomyelitis is an epidemic-prone vaccine-preventable disease (VPD) that causes disabilities due to the effects of the poliovirus [1,2]. Recent reports indicate that, of the 6 World Health Organization (WHO) Regions, only the Eastern Mediterranean Region (EMR) still has countries reporting wild poliovirus (Afghanistan and Pakistan). The other five regions were certified free of the indigenous wild poliovirus. The polio endgame strategy 2019-2023 addresses three goals; eradication, integration, and certification. The acute flaccid paralysis (AFP) surveillance is one of the key interventions for the implementation of the three goals of the polio strategic plan [3]. The main clinical presentation of poliomyelitis is acute flaccid paralysis, and therefore AFP surveillance is the gold standard for polio eradication and post eradication [4]. Nevertheless, most countries currently are implementing or planning to implement environmental surveillance to supplement the AFP surveillance after realizing its benefit towards global polio eradication [5-7]. Interestingly, the use of sewage to supplement public health surveillance of infectious diseases based on the experience of poliomyelitis surveillance, offers other opportunities for detecting other infectious diseases of public concern such as the SARS-CoV-2 which has been initiated in Japan [8,9]. The WHO East and Southern Africa (ESA) sub region serves the following countries; Botswana, Comoros, Eritrea, Eswatini, Ethiopia, Kenya, Lesotho, Madagascar, Malawi, Mauritius, Mozambique, Namibia, Rwanda, Seychelles, South Africa, South Sudan, Tanzania, Uganda, Zambia, and Zimbabwe. All countries in the ESA sub-region are implementing AFP surveillance. Additionally, 9 of the countries implement environmental surveillance to increase the poliovirus detection sensitivity of the AFP surveillance system. In the WHO Afro region, Algeria was the first country to report a confirmed COVID-19 case caused by severe acute respiratory syndrome Coronavirus 2 (SARS-CoV-2) on 25^th^ February 2020. In the East and Southern Africa sub-region, South Africa confirmed the first case on 5^th^ March 2020, almost three months after the initial isolation of the coronavirus on 7^th^ January 2020 in Wuhan China [10-12]. From March to April 2020, disruptions of routine vaccinations in 129 countries were experienced including complete cessation in some, postponing preventive and outbreak response 91 vaccination campaigns in the initial 5 months of the COVID-19 pandemic [13]. In turn, the VPDs surveillance was also affected by the disruption of services, lockdown measures, and missed opportunities of vaccination campaigns to reach more communities. Additionally, the change of focus posed by the COVID-19 pandemic (by the governments, partners, and health workers) and the disruption of vaccination services resulted in growing fear of infecting health care providers and clients which negatively impacted on VPDs surveillance operations [14].

A study in England found that physical distancing amidst the COVID-19 pandemic reduced the infant measles-mumps-rubella vaccination counts by 19.8% (95% CI, ranges from -20.7 to -18.9) in 3 weeks period compared to the 2019 figures [15]. The review of vaccination data from Michigan state immunization system involving cohorts from 2016 to 2020 for the period january to may indicated that vaccination for all age cohorts declined from 2016 to 2019 to 2 quarter (ranges 66.6% in 2016 to 67.9% in 2019), and dramatic decline in 2020 to 47.9% [16]. In this regard, the disruption of infant vaccination services equally affects the vaccine's preventable surveillance services, as they are all being delivered in the same package and by the same person at the service provisional points. A modeling study using the lives saved tool (LiST) conducted in 2020 for the low and middle-income countries on maternal and child mortality indicated that, there is a reduction of essential maternal and child health interventions coverage (including EPI services) by 9-8-51.9% following the COVID-19 pandemic [17]. A study was conducted in Pakistan to evaluate the impact of the COVID-19 pandemic lockdown for a period before (6 months before lockdown) and during the lockdown for the children registered in the Zindagi Mehfooz Electronic immunization registry in Karachi. The study revealed a 52.8% reduction of the mean number of daily all antigens vaccination visits (from 5,184 to 2,450 visits) and, 48 health facilities were closed in the study area [18]. Additionally, in the study of the 321 operational centers, 50 (16%) had no clients who feared contacting the COVID-19 while 271 vaccination centers 48 (18%) were closed due to disruption of supplies. In this regard, because of fear, lockdown measures, and disruption of supplies AFP surveillance could be equally impacted by the COVID-19 pandemic. The supply disruption amidst the COVID-19 pandemic was projected based on the previous diseases epidemics and indicated the delayed deliveries of supplies, which include delays in stool specimen shipment also which are important in implementing AFP surveillance [19]. On 20^th^ June 2020, WHO, United Nations Children’s Fund (UNICEF), and Gavi, in collaboration with other global immunization stakeholders, conducted a second poll involving 260 participants from 82 countries to assess the disruption of vaccination services caused by the COVID-19 pandemic and the lockdown measures. The polls revealed that in the WHO/AFR region, 44% of the countries had restricted fixed vaccination posts, 72% of countries had also disrupted outreach vaccination services, and 14% of countries canceled all outreach vaccination services [20]. These results also reflect challenges that contributed to the same countries for AFP surveillance amidst the COVID-19 pandemic. The WHO/AFR region certified as free of the indigenous wild poliovirus on 25^th^ August 2020. The polio-free certification is among the achievements of global polio eradication. However, the challenge posed by the COVID-19 pandemic such as widespread lockdown, the fears, and social distancing measures adversely affected the AFP surveillance and the Global polio eradication milestones in general. We, therefore, decided to conduct an assessment of the early effects of the COVID-19 pandemic on AFP performance in the ESA sub-region for the evidenced documentation. Findings of the early effects will also contribute to the overall general situation analysis review to aid in the resumption of AFP surveillance and catch-up of postponed the activities due to the COVID-19 pandemic.

## Methods

**Study design:** we conducted comparative analysis of secondary AFP surveillance data from the East and Southern Africa sub-region to evaluate the effect of the COVID-19 pandemic on AFP field surveillance. The review of monthly surveillance quality performance indicators was done from March to December of 2019 and 2020 keeping in mind the respective pre and post COVID19 setting. Acute flaccid paralysis field surveillance indicators were used starting from the case notification to receipt of the stool specimens in the polio laboratories. This was to evaluate the sensitivity of the surveillance systems. Furthermore, we compared the 2019 and 2020 findings at ESA global sub regional and country levels. Additional information from the Global Polio Laboratory Network data bases was processed for the same period to understand the condition of the specimens at the time of receipt in the polio laboratories. The monthly reported AFP surveillance performances was compared for the two years. Where variation was noted, the differences were subjected to statistical tests for ascertaining levels of significance.

**Study population:** the study involved all reported AFP cases, as per clinical diagnosis, with onset of paralysis from 1^st^ March 2019 to 31^st^December 2020 from 19 of the 20 ESA countries namely Botswana, Comoros, Eritrea, Ethiopia, Eswatini, Lesotho, Kenya, Madagascar, Malawi, Mauritius, Mozambique, Namibia, Rwanda, South Africa, South Sudan, Tanzania, Uganda, Zambia, and Zimbabwe. The Seychelles was excluded from the study because the country reported only 1 AFP case in the past ten years and therefore would not provide meaningful comparative effect of the COVID-19 pandemic. The WHO standard case definition for AFP was used in the study. An AFP case was defined as a child less than 15 years of age presenting with recent or sudden onset of floppy paralysis or muscle weakness due to any cause, or any person of any age with paralytic illness if poliomyelitis is suspected by a clinician. All AFP cases with circulating vaccines derived polioviruses and those reported as compatible with polio were included in the study. Not all performance indicators were enlisted. All AFP cases that were finally discarded due to classification as “Not AFP” were excluded from the study.

**Key parameters:** the WHO standard key surveillance indicators were used as parameters to assess the effects of the COVID-19 pandemic on the AFP surveillance systems. The scope of parameters covered from the notification of the reported AFP cases to the release of results by the WHO accredited polio laboratories. For the completeness and timeliness of routine AFP surveillance reports, we used the percentage of designated sites reporting AFP data, even in the absence of cases, and the percentage of designated surveillance sites reporting AFP data on time (even in the absence of cases). The Non-polio AFP (NPAFP) rate was used to assess the sensitivity of the surveillance systems and the percentage of AFP cases reported to health authorities in less than seven days from the onset of paralysis for the timeliness of notification. The timeliness of investigation of the reported AFP cases was measured by the percentage of the AFP cases investigated within 48 hours of notification while the adequate stool specimen collection assessed by the percentage of AFP cases with two stool specimens collected 24 hours apart (both within 14 days of paralysis onset, and the receipt of these specimens in good condition at the WHO accredited national polio laboratory). The completeness of 60-day follow-up was assessed by the percentage of AFP cases with a follow-up examination conducted for residual paralysis at 60 days after the onset of paralysis and the proportion of specimens arriving at a WHO accredited laboratory within three days of collection was used to evaluate the timeliness of stool specimen´s shipment.

**Data collection and analysis:** we retrieved the weekly AFP surveillance data from the AFP database in the Global Polio Eradication Initiative, the polio information system of March to December in 2019 and 2020 for the WHO ESA sub region countries. Descriptive analysis was done using Epi info version 3.5.4 supplemented by pivot table functions of Microsoft Excel and the SPSS version 22 for the inferential statistics. Weekly and monthly data series used were based on the study assumption that lockdown measures and social distancing kept changing on weekly and or monthly basis depending on the COVID19 transmission progression patterns.

## Results

A total of 9,914 acute flaccid paralysis cases were reported during the study period of March to December of 2019 and 2020. The majority of the paralyzed cases under 15 years population were men (54%), and the remaining 45% were female (Table 1). The sex characteristic was missing in 1% of the study population in the database. Males were more likely to have AFP and this observation constituted a nine-percentage change in the study period with a statistical significance difference p-value of less than 0.0001 (95% CI ranges from 7% to11%) and the t-statistics value of 71.285. The most affected age group was the under-five children that involved 5,648 (57%) of the total study population. We observed a 26.7% change between the reported AFP cases from the under five years of age and 5 to 14 years aged children. This difference was statistically significance with a p-value of less than 0.0001 (95% CI, ranges from 24.587% to28.767%). Also, a total of 4,336 children aged 0 -3 years old paralyzed during the study period contributed 43.7% of the total AFP cases reported Table 1. The same age group contributed to 2,320 children which was 43.4% before the COVID-19 pandemic (March - December 2019) and 2,016 children (43.9%) from March to December 2020. The children aged 5 to 14 years were 2,999 (30.3%), while the least affected age group was that of over 15 years which contributed only 1% (60 people). Out of all the reported patients with AFP 7,130 (71.9%) presented with fever at the onset of paralysis. From March to December 2019, 3,865 AFP cases (72.6%) presented with fever compared to 3,265 AFP cases (71.1%) in 2020 (Table 2). The 1.5% change between the two years was not statistically significant (p=0.1603, 95% CI ranges from -0.592 to 3.599). We also observed monthly variation, being lowest in April 2020 (5.79%) and highest in October 2020 (14.33%). We compared the findings of the lowest and highest results for April and October 2020 and observed a significant difference in October 2020 only (p-value of 0.018, 95% CI ranges from 0.956 to 9.795).

**Table 1:** characteristics of the reported acute flaccid paralysis cases and suspected poliomyelitis cases, March -December 2019 and 2020 in East and Southern Africa countries

Parameters	Year (n)	Mar	Apr	May	Jun	Jul	Aug	Sep	Oct	Nov	Dec
# of reported AFP cases	2019 (n= 5,322)	10.9	10.1	11	10.2	10.7	11	9.79	9.08	10.1	7.2
# of reported AFP cases	2020 (n= 4,592)	9.54	5.9	8.69	8.62	9.86	10.9	12.2	14.7	11.9	7.6
% of Females	2019 (n=2,370)	10.9	10.1	11.8	10.3	10.2	10.5	9.62	9.2	10.4	6.9
% of Females	2020 (n=2,099)	9.67	5.96	9.43	8.91	10.1	10.2	11.1	15.1	11.4	8.1
% of Males	2019 (n=2,880)	11.2	9.9	10.2	10.2	11.2	11.4	10	8.85	9.79	7.3
% of Males	2020 (n=2,452)	9.38	5.87	8.08	8.36	9.62	11.5	13.2	14.4	12.4	7.1
% of Age < 5 years	2019 (n=2,994)	10.8	10.1	10.4	10.2	11	11.2	10.1	8.78	10.6	7
% of Age < 5 years	2020 (n=2,654)	8.74	5.24	8.48	8.93	10.1	9.8	12.2	15.5	13.3	7.6
% of Age: 5 - 14 years	2019 (n=1,614)	10.7	10.5	11	9.54	9.42	10.5	9.67	10	11.2	7.5
% of Age: 5 - 14 years	2020 (n=1,385)	10.8	6.64	9.89	7.87	9.17	12.4	12.3	12.9	10.7	7.3
% of Age: >15 years	2019 (n=32)	21.9	9.38	21.9	15.6	9.38	0	9.38	3.13	9.38	0
% of Age: >15 years	2020 (n=28)	14.3	17.9	7.14	7.14	7.14	10.7	7.14	21.4	7.14	0

**Table 2:** clinical presentations of the reported acute flaccid paralysis cases and suspected poliomyelitis cases, March -December 2019 and 2020 in East and Southern Africa countries

Clinical presentations	Year (n)	Mar	Apr	May	Jun	Jul	Aug	Sep	Oct	Nov	Dec
% of the reported AFP cases presented with fever at the onset of paralysis	2019 (n=3,865)	11.38	10.45	10.97	10.74	11.10	10.22	9.13	8.85	10.17	6.99
	2020 (n=3,265)	9.59	5.79	9.00	8.94	10.20	10.23	12.10	14.33	11.91	7.90
% of the reported AFP cases where the progression of paralysis was within 3 days (Acute)	2019 (n=3,545)	11.28	9.68	11.06	10.52	11.42	11.37	9.56	9.11	9.31	6.69
	2020 (n=3,091)	8.99	5.40	8.38	8.70	10.16	11.32	11.78	14.75	12.49	8.02
% of the reported AFP cases presented with asymmetrical paralysis	2019 (n=2,284)	11.65	10.46	11.73	11.21	10.95	11.51	8.71	8.27	9.37	6.13
	2020 (n=2,053)	8.77	5.07	7.60	8.87	10.08	11.15	11.54	15.29	13.35	8.28
% of the reported AFP cases presented with paralysis of lower limb(s).	2019 (n= 4,772)	11.25	9.87	10.96	10.27	10.79	11.19	9.87	8.72	9.81	7.27
	2020 (n= 4,173)	9.35	5.82	8.79	8.75	9.87	10.74	12.41	14.91	11.91	7.45

In respect to lower-limb paralysis, a total of 8,945 (90.2%) of the reported AFP cases had paralysis from March 2019 to December 2020, and the majority (53%) were in March to December 2020. Furthermore, we observed monthly variations over the study period, being lowest from March to July 2020 compared to the same period in 2019. The lowest peak was in April 2020 (5.82%) and the highest peak (14.91%) in October 2020. We observed significant changes for the highest peak of lower-limb paralysis with a p-value of 0.0056 (95% CI ranges from 1.817 to 10.534). Additionally, 66.9% of the reported AFP cases had sudden onset (progressing within 3 days) and 43.7% had asymmetrical paralysis Table 2. A total of 7,348 of the reported AFP cases were notified within 7 days, reflecting 74.1%. From March to December 2019, a total of 3,939 (74.4%) reported AFP cases were notified within 7 days of the onset of paralysis compared to 3,389 (73.8%) AFP cases from March to December 2020 (Table 3). A decline in the notification of 0.6% was observed in 2020 from the March to December 2019 cases. There was no statistically significant difference; p-value of 0.496 (95% CI, ranges from -1.126 to 2.332). As well, a monthly variation was observed in the notification being highest was in October 2020 (14.16) and lowest in April 2020 (6.37). We observed a 5.45% increase in the onset of paralysis to the notification of AFP cases for October 2020 compared to October 2019. The difference was statistically significant, with a p-value of 0.017 (95% CI, ranges from 0.986 to 9.697). Furthermore, a total of 8,926 (90%) of the reported AFP cases in the study period were investigated within 48 hours of notification. From March to December 2019, a total of 4,819 (90.5%) of the reported AFP cases were investigated within 48 hours and 4,107 (89.4%) in 2020 while there was a 1% decline in 2020 that was not statistically significant; p-value of 0.099 (95% CI), ranges from -0.187 to 2.198). A total of 4,995 (50.4%) of the second stool specimens collected from the reported AFP cases were delivered to the polio laboratories within 3 days (72 hours) (Table 4). A high proportion (52.4%) of the second stool samples were received in the laboratory within 72 hours from March to December 2019, while a lower proportion (48.1%) was received in the same time period in 2020. We observed a 4.3% decrease in 2020, a statistically significant change with a p-value of <0.0001 (95% CI, ranges from 2.326% to 6.269%). Also, a total of 6,164 (62.1%) of the reported AFP cases stool's specimens were delivered within 7 days after collection into the polio laboratories. A higher proportion (64.3%) was again was delivered from March to December of 2019, compared to 59.6% delivered in March to December 2020. We noticed a statistical significance change with a p-value of less than 0.001 (95% CI, ranges from 2.784% to 6.614%). The monthly variation of the reported AFP cases existed. For April 2020 the AFPs second stool specimen collected and delivered into polio laboratories within 3 days for the study period was 3.9%, while in October 2020 it was 17.49% Table 4. We compared the proportion of the reported AFP cases stool specimens delivered in the laboratory for more than 30 days from March to December 2019 (3.2%) and in the same period of 2020 (7.8%). We observed a 4.6% increase from March to December 2020 compared to the same time period in 2019. A statistical significance of a p-value of less than 0.001 was observed (95% CI ranges from 3.702 to 5.523).

**Table 3:** timeliness of reported AFP cases from onset of paralysis to the field investigation of the cases, March -December 2019 and 2020 in East and Southern Africa countries

Parameters	Year (n)	Mar	Apr	May	Jun	Jul	Aug	Sep	Oct	Nov	Dec
% of specimens delivered in lab within 3 days	2019 (n=2,788)	10.69	10.15	10.40	8.86	9.58	10.87	10.44	10.62	10.58	7.82
	2020 (n=2,207)	9.42	3.90	7.20	7.20	7.66	9.20	13.55	17.49	14.77	9.61
% of specimens delivered in lab within 7 days	2019 (n=3,427)	10.65	10.24	10.15	8.67	9.86	10.88	10.36	10.85	10.68	7.65
	2020 (n=2,737)	9.06	4.86	8.37	7.23	8.51	9.61	13.01	16.70	13.85	8.81
% of specimens delivered in lab more than 30 days	2019 (n=172)	13.37	8.14	13.37	8.72	10.47	8.72	6.40	12.79	9.88	8.14
	2020 (n=368)	18.75	14.67	13.04	13.32	15.76	11.68	9.78	1.90	1.09	0.00
% of specimens delivered in lab with good condition	2019 (n=4,710)	11.32	10.45	10.96	10.49	10.74	11.38	10.15	8.62	9.62	6.28
	2020 (n=4,292)	9.11	5.58	8.37	7.96	9.02	9.70	10.83	13.59	11.15	5.82

**Table 4:** timeliness of reported AFP cases from the collection of second stool to the delivery of stool specimens in polio laboratories, March -December 2019 and 2020 in East and Southern Africa countries

Parameters	Year (n)	Mar	Apr	May	Jun	Jul	Aug	Sep	Oct	Nov	Dec
% of specimens delivered in lab within 3 days	2019 (n=2,788)	10.69	10.15	10.40	8.86	9.58	10.87	10.44	10.62	10.58	7.82
	2020 (n=2,207)	9.42	3.90	7.20	7.20	7.66	9.20	13.55	17.49	14.77	9.61
% of specimens delivered in lab within 7 days	2019 (n=3,427)	10.65	10.24	10.15	8.67	9.86	10.88	10.36	10.85	10.68	7.65
	2020 (n=2,737)	9.06	4.86	8.37	7.23	8.51	9.61	13.01	16.70	13.85	8.81
% of specimens delivered in lab more than 30 days	2019 (n=172)	13.37	8.14	13.37	8.72	10.47	8.72	6.40	12.79	9.88	8.14
	2020 (n=368)	18.75	14.67	13.04	13.32	15.76	11.68	9.78	1.90	1.09	0.00
% of specimens delivered in lab with good condition	2019 (n=4,710)	11.32	10.45	10.96	10.49	10.74	11.38	10.15	8.62	9.62	6.28
	2020 (n=4,292)	9.11	5.58	8.37	7.96	9.02	9.70	10.83	13.59	11.15	5.82

In the ESA sub-region, the Non-Polio AFP rate declined from 3.3 (March to December 2019) to 3 per 100 000 population aged less than 15 years in the same period in 2020 (Table 5). We observed a decline of 0.3 in 2020, and no statistical difference was seen, a p-value of 1.000 (95% CI, ranges from -0.323 to 0.227) and t-statistics of 25.962. However, we noticed a 4.7% decline in the number of reported AFP cases in the ESA sub-region for March to December 2020 and the same period in 2019 and this was statistically significant with a p-value of less than 0.001 (95% CI ranges from 2.785 to 6.614). A decline of the non-polio AFP rate per 100 000 population aged less than 15 years from March to December 2020 was observed 13 of the 19 assessed ESA countries in; Botswana, Comoros, Eswatini, Kenya, Madagascar, Malawi, Mauritius, Mozambique, Namibia, Rwanda, South Africa, Uganda, and Zimbabwe. Nevertheless, Eritrea, Ethiopia, Lesotho, South Sudan, Tanzania, and Zambia reported an increase in the non-polio AFP rate in 2020 compared to 2019. More decline was observed in Mauritius, Botswana, South Africa, and Mozambique. The highest increase was observed Eritrea, South Sudan, and Zambia. Also, the monthly variation of the non-polio AFP rate for the ESA sub region, March to December 2019, and 2020 data indicate a decline in 2020 from March to August 2020. The highest decline was in April 2020 by 1.7 (50% decline) and an increase peaked by October 2020 reflecting 1.4 (45% increase). The decrease we observed in April and an increase in October were not statistically significant in non-polio AFP rates. In the study period, the percentage of stool adequacy average from March to December 2019 was 90% and 89% for the same period in 2020 in the ESA sub-region (Table 6). This indicates a 1% decline that is not statistically significant, a p-value of 0.105 (95% CI ranges from -0.208 to 2.218). The country variation existed in Comoros, Eswatini, Kenya, Madagascar, Malawi, Namibia, South Africa, South Sudan, and Zambia indicated a decline in the percentage of stool adequacy from March to December 2020 when compared to 2019 figures. Surprisingly, the percentage of stool adequacy increased in 2020 compared to 2019 in Botswana, Mozambique, Rwanda, Uganda, and Zimbabwe. We noticed a monthly variation of the stool adequacy for the ESA sub region, the lowest being in April 2020 (85.7% reflected in the table as of 86%) and highest in September 2019 (93%). We observed a 3.37% decline for April in 2020 compared to April 2019, a statistically significant difference with a p-value of less than 0.001 (95% CI ranges from 2.059 to 4.690). Also, in September, we saw a 6% decline in 2020, statistically significant with a p-value of less than 0.001 (4.817% to 7.198%). The proportion of the under-five children from the reported AFP cases, who received less than three Oral poliovirus vaccines (OPV) doses increased from 21.8% in March to December 2019 to 24.9% in the same period in 2020. We observed a 3.01 percentage change, which is statistically significant with a p-value of 0.0001 (95% CI, ranges from 1.502% to 4.519%). Also, country variations were seen where the increase in the proportion of under-fives vaccinated with less than 3 doses from the reported AFP cases was high in Ethiopia (18%) and low in Kenya (2.5%) when comparing figures for 2019 and 2020.

**Table 5:** the monthly non polio AFP rate for children aged less than 15 years by countries, March - December 2019 and 2020, East and Southern Africa

Countries	Year	Mar	Apr	May	Jun	Jul	Aug	Sep	Oct	Nov	Dec	Mean
Botswana	2019	3.1	1.5	3.1	1.5	3.1	3.1	3.1	3.1	7.6	6.1	3.5
	2020	6.1	3.1	1.5	3.1	0.0	1.5	0.0	0.0	0.0	0.0	1.5
Comoros	2019	3.2	0.0	6.4	3.2	0.0	3.2	0.0	9.6	6.4	0.0	3.2
	2020	0.0	0.0	0.0	0.0	4.3	2.2	13.0	2.2	4.3	4.3	3.0
Eritrea	2019	7.2	12.9	12.9	8.6	3.6	7.9	6.5	5.0	3.6	0.0	6.8
	2020	5.0	3.6	8.6	15.1	12.2	20.8	13.6	7.9	7.2	9.3	10.3
Ethiopia	2019	2.5	2.4	2.4	2.2	2.2	3.5	2.9	3.1	3.5	3.5	2.8
	2020	2.4	0.6	1.6	1.2	2.0	2.4	4.5	6.8	5.4	3.1	3.0
Kenya	2019	3.7	2.8	2.5	2.8	2.7	2.6	2.5	2.6	1.9	1.7	2.6
	2020	1.4	0.7	1.2	1.6	2.0	1.7	2.0	1.6	1.9	0.7	1.5
Lesotho	2019	3.8	7.5	5.6	0.0	1.9	1.9	0.0	0.0	5.6	0.0	2.6
	2020	5.6	0.0	1.9	0.0	1.9	3.8	1.9	3.8	3.8	9.4	3.2
Madagascar	2019	5.6	8.9	5.1	4.4	6.0	8.0	6.3	4.5	6.2	4.5	5.9
	2020	3.9	5.8	10.0	4.5	4.9	4.7	6.0	7.1	6.0	4.4	5.7
Malawi	2019	2.1	2.1	2.4	3.7	2.7	2.4	1.6	2.4	2.0	1.3	2.3
	2020	1.3	2.3	2.1	2.1	0.6	1.3	1.3	2.0	1.9	1.4	1.6
Mauritius	2019	5.1	0.0	0.0	0.0	5.1	10.3	5.1	0.0	0.0	10.3	3.6
	2020	0.0	0.0	0.0	5.1	0.0	5.1	5.1	0.0	0.0	0.0	1.5
Mozambique	2019	3.6	3.9	5.3	8.5	6.7	5.2	4.0	2.6	2.1	2.1	4.4
	2020	2.2	1.2	2.6	3.7	5.0	5.3	3.3	3.9	3.1	1.4	3.2
Namibia	2019	0.0	1.2	3.7	1.2	1.2	2.5	1.2	2.5	4.9	3.7	2.2
	2020	2.5	1.2	2.5	0.0	0.0	0.0	2.5	0.0	1.2	0.0	1.0
South Sudan	2019	10.7	8.4	6.9	7.4	7.2	5.5	4.6	3.4	3.8	1.9	6.0
	2020	8.6	5.7	6.1	5.5	6.7	7.1	9.5	8.6	7.4	3.8	6.9
Rwanda	2019	0.8	0.8	3.1	1.3	3.1	1.6	3.7	5.2	4.7	2.6	2.7
	2020	1.8	0.3	1.6	1.6	1.6	0.0	3.4	2.6	2.9	5.0	2.1
South Africa	2019	4.2	4.0	4.9	2.5	3.5	3.9	3.8	3.9	3.3	2.2	3.6
	2020	4.0	2.4	1.4	1.5	1.5	1.9	1.3	2.8	3.2	1.9	2.2
Eswatini	2019	11.3	2.8	0.0	5.7	2.8	5.7	5.7	0.0	8.5	0.0	4.2
	2020	2.8	0.0	2.8	2.8	5.7	0.0	5.7	0.0	2.8	8.5	3.1
Tanzania	2019	4.0	2.6	4.1	3.5	4.1	3.5	3.0	2.1	4.1	3.2	3.4
	2020	3.8	1.1	2.1	3.4	3.8	4.6	4.1	5.0	4.8	3.9	3.7
Uganda	2019	3.6	3.3	3.3	3.1	3.2	2.3	1.9	2.3	2.7	0.8	2.7
	2020	1.3	1.1	1.9	1.4	1.8	2.2	1.9	2.7	2.8	3.7	2.1
Zambia	2019	1.4	0.7	2.3	2.5	2.9	2.9	4.5	5.2	3.8	1.6	2.8
	2020	3.0	3.9	2.4	5.7	5.7	4.5	3.1	3.7	3.6	2.2	3.8
Zimbabwe	2019	4.6	2.5	3.8	2.9	3.5	2.1	4.0	2.3	2.5	0.2	2.8
	2020	2.7	1.4	3.1	3.1	1.9	2.3	3.3	4.3	1.9	1.0	2.5
ESA sub-region	2019	3.6	3.4	3.7	3.4	3.6	3.6	3.3	3.1	3.4	2.4	3.3
	2020	2.8	1.7	2.5	2.5	2.9	3.2	3.6	4.5	3.9	2.8	3.0

**Table 6:** the percentage of monthly stool of the reported AFP cases by countries, March - December 2019 and 2020, East and Southern Africa

Country	Year	Mar	Apr	May	Jun	Jul	Aug	Sep	Oct	Nov	Dec	Average
Botswana	2019	100%	100%	50%	0%	100%	50%	50%	50%	80%	25%	61%
	2020	75%	100%	0%	50%	N/A	100%	N/A	N/A	N/A	N/A	65%
Comoros	2019	100%	N/A	100%	100%	N/A	100%	N/A	100%	100%	N/A	100%
	2020	N/A	N/A	N/A	N/A	100%	100%	83%	100%	50%	0%	72%
Eritrea	2019	100%	100%	100%	100%	100%	100%	100%	100%	100%	N/A	100%
	2020	100%	100%	100%	100%	100%	100%	100%	100%	100%	100%	100%
Ethiopia	2019	88%	93%	91%	87%	91%	91%	95%	89%	89%	93%	91%
	2020	95%	100%	93%	93%	94%	85%	75%	92%	88%	95%	91%
Kenya	2019	89%	94%	90%	96%	93%	91%	98%	98%	90%	93%	93%
	2020	96%	75%	89%	81%	91%	83%	72%	82%	82%	83%	84%
Lesotho	2019	100%	100%	100%	N/A	100%	100%	N/A	N/A	100%	N/A	100%
	2020	100%	N/A	100%	N/A	100%	100%	100%	100%	100%	100%	100%
Madagascar	2019	85%	94%	96%	95%	98%	99%	98%	93%	98%	93%	95%
	2020	86%	89%	95%	88%	96%	87%	95%	96%	95%	90%	92%
Malawi	2019	80%	73%	88%	96%	89%	88%	91%	82%	93%	89%	87%
	2020	100%	88%	80%	100%	75%	56%	100%	93%	83%	100%	87%
Mauritius	2019	100%	N/A	N/A	N/A	100%	100%	100%	N/A	N/A	100%	100%
	2020	N/A	N/A	N/A	100%	N/A	100%	100%	N/A	N/A	N/A	100%
Mozambique	2019	76%	61%	82%	54%	81%	80%	79%	81%	82%	59%	74%
	2020	71%	71%	86%	83%	79%	87%	82%	60%	73%	83%	78%
Namibia	2019	N/A	100%	100%	0%	100%	100%	100%	100%	75%	100%	86%
	2020	50%	0%	50%	N/A	N/A	N/A	100%	N/A	100%	N/A	60%
South Sudan	2019	88%	93%	94%	95%	84%	90%	96%	78%	90%	90%	90%
	2020	91%	97%	66%	83%	83%	89%	90%	80%	92%	N/A	86%
Rwanda	2019	100%	100%	100%	100%	92%	67%	100%	70%	94%	90%	91%
	2020	100%	100%	83%	100%	100%	N/A	100%	70%	100%	79%	92%
South Africa	2019	90%	83%	79%	86%	86%	93%	87%	88%	87%	88%	87%
	2020	91%	79%	86%	81%	73%	90%	89%	85%	83%	71%	83%
Eswatini	2019	75%	100%	N/A	100%	100%	100%	100%	N/A	100%	N/A	96%
	2020	100%	N/A	100%	100%	100%	N/A	100%	N/A	0%	100%	86%
Tanzania	2019	96%	96%	98%	96%	99%	99%	100%	95%	99%	97%	97%
	2020	99%	95%	98%	100%	100%	99%	99%	96%	96%	96%	98%
Uganda	2019	86%	84%	90%	96%	91%	85%	88%	90%	91%	93%	90%
	2020	96%	84%	97%	92%	88%	97%	88%	86%	86%	92%	91%
Zambia	2019	80%	100%	88%	82%	70%	85%	87%	83%	81%	82%	84%
	2020	70%	67%	56%	65%	70%	67%	79%	73%	N/A	N/A	68%
Zimbabwe	2019	91%	100%	78%	86%	100%	70%	89%	73%	92%	100%	88%
	2020	77%	86%	87%	80%	89%	91%	100%	90%	100%	100%	90%
ESA sub-region	2019	88%	89%	90%	86%	91%	91%	93%	88%	91%	90%	90%
	2020	91%	86%	88%	88%	89%	89%	87%	88%	89%	91%	89%

## Discussion

A decline was in the number of reported AFP cases in the March to December period 2020 compared to the same period in 2019. This indicated the effects of the COVID-19 prevention measures such as quarantine, lockdown, physical/social distancing, international travel restrictions, and others on the AFP surveillance in the ESA sub-region. These lockdown and transport restrictions effects equally affected the environmental surveillance performances where samples have to be transported inside and outside the countries. Interestingly, we observed that the AFP cases reported with fever at onset of paralysis from March to December 2020 declined compared to the same time period in 2019. This may be due to the fear that fever is a suspect of the COVID-19 and therefore was bound to be misdiagnosed, but also due to decline in the precision diagnosis due to limited or no physical clinician sensitization, supportive supervision, and mentorship in most of the countries. The period between the onset of the paralysis and notification for the reported AFP cases showed a 0.6% decline in March to December 2020 compared to the same period in 2019. However, monthly variation in April and October indicated a significant difference. We observed a decline in April 2020 contributed by the effects of the COVID-19 disease prevention measures. In October 2020, the availability of clear guidelines and information to countries on the mode of transmission of the COVID-19 helped to uplift some measures in favor of AFP field surveillance. Additionally, the WHO interim guidelines of continuation of the immunization services and VDPs surveillance were implemented by several countries [21-23]. We found that only 50.4% of the collected stool specimens from the reported AFP cases were delivered in laboratories within 72 hours which was far much lower than the 80% minimum target, showing a 29.6-percentage difference. This target is practically difficult to achieve in ESA countries that do not host polio laboratories, considering the time to deliver the stool specimens from the field to the national level and thereafter shipped by a courier agent to the intercountry polio laboratory following a standard WHO guideline [24]. Concerning the COVID-19 pandemic environment, we compared from March to December in 2019 and 2020, a further 4.2% decline in 2020 was contributed by the lockdown measures and international shipment restrictions that affected countries using intercountry laboratories. Even by extending from 3 to 7 days delivery time frame (from the collection of second stool from reported AFP cases), the proportion of specimens delivered to polio laboratories was 62.1%. A 17.15% difference from the 80% benchmark. There was a 4.7% change by comparing the 2019 and 2020 stool specimen deliveries in the laboratories with the 7 days´ time frame. A further decline in the proportion of stool samples shipped within 72 was seen in April 2020 compared to the same month in 2019. These delays of stool specimen shipment transportation from field to laboratory amidst the COVID-19 pandemic and batching could have been worse where extra active corrective initiatives were not implemented in the ESA countries. Among the effective interventions were the weekly feedback on stool tracking and the online weekly stool specimen monitoring.

## Conclusion

The COVID-19 pandemic impacted AFP surveillance in the East and Southern African countries in notification, investigation, and shipment of stool samples to the laboratories, releasing laboratory results, and therefore delayed detection of possible outbreaks and continued circulation. Additionally, the quality of AFP surveillance was affected due to lack of physical/reduced clinician orientations, surveillance mentorship, and physical supportive supervision. However, the use of technology-supported the continuation of AFP surveillance and to achieve the minimum requirements by the end of 2020. Further specific country studies are advised to assess detail at the sub-national level to the countries indicating the substantial decline of the core AFP surveillance indicators. The decline of the core AFP surveillance, NP-AFP rate, and percentage of stool adequacy shown in most countries that implemented complete lockdown, and a slight increase in countries where lockdown measures were not stringent. The overall decline of AFP cases from March to December 2019 to the same period in 2020 in the ESA sub-region indicated a 7.4% decline which was statistically significant. The major decline was noted in March to August 2020, the raised in August followed the release of WHO guidelines on AFP surveillance in the COVID-19 pandemic context, remote AFP surveillance checklist, and most countries relaxed the COVID-19 pandemic restrictive measures. Similarly, as a reflection of the disruption of childhood vaccination and an increase in the number of incomplete vaccinations was observed in 2020 from the reported AFP cases. The result may contribute to the emergence of Vaccine-derived poliovirus (VDPVs) and delayed interruption of ongoing circulating Vaccine-derived poliovirus (cVDPV2) due to inadequate response activities.

### What is known about this topic

- The COVID-19 pandemic adversely impacted operations of AFP and environmental surveillance in the ESA sub-region due to the disruption of supplies, implementation lockdown, and social distancing;
- The East and Southern Africa sub-region WHO office supported the ESA countries amidst the COVID-19 in the deployment of remote surveillance checklist and virtual capacity building;
- The AFP surveillance in some countries in the ESA sub-region was integrated with the COVID-19 pandemic surveillance, the repurposing of polio eradication human and other resources creating a gap for polio eradication initiatives including polio specific and other VPD disease surveillance.

### What this study adds

- The study identifies specific areas in which AFP surveillance was affected and recommend ways for improvement during the pandemic and after the resumption of services post the COVID pandemic;
- It provides invaluable information from the ESA countries that can be used by the Global Polio Eradication and being used to support other countries in WHO/AFRO or other WHO regions;
- The need for scaling up the user experiences of virtual and remote strategies in the COVID-19 pandemic context like zoom meetings to reduce future programmatic operational costs is elaborated, it reinforces the value of integrating continuity of essential services with response to emerging and re-emerging diseases.
